# *Prosopis africana* exerts neuroprotective activity against quaternary metal mixture-induced memory impairment mediated by oxido-inflammatory response via Nrf2 pathway

**DOI:** 10.3934/Neuroscience.2024008

**Published:** 2024-04-22

**Authors:** Orish E. Orisakwe, Evelyn Utomoibor Ikpeama, Chinna N. Orish, Anthonet N. Ezejiofor, Kenneth O. Okolo, Aleksandar Cirovic, Ana Cirovic, Ify L. Nwaogazie, Chinekwu Samson Onoyima

**Affiliations:** 1 African Centre of Excellence for Public Health and Toxicological Research (ACE-PUTOR), University of Port Harcourt, PMB, 5323 Port Harcourt, Choba, Nigeria; 2 Advanced Research Centre, European University of Lefke, Lefke, Northern Cyprus, TR-10 Mersin, Turkey; 3 World Bank Africa Centre of Excellence in Oilfield Chemicals Research (ACE-CEFOR), University of Port Harcourt, PMB, 5323 Port Harcourt, Choba, Nigeria; 4 Department of Anatomy, Faculty of Basic Medical Sciences, College of Health Sciences, University of Port Harcourt, PMB, 5323 Port Harcourt, Choba, Nigeria; 5 Department of Pharmacology & Toxicology, Faculty of Pharmacy, Enugu State, University of Science & Technology, Nigeria; 6 University of Belgrade, Faculty of Medicine, Institute of Anatomy, Belgrade, Serbia; 7 Dept. of Biochemistry, Faculty of Biological Sciences, University of Nigeria Nsukka, Enugu State, Nigeria

**Keywords:** *Prosopis africana*, heavy metals, neuroprotective effects, cerebral cortex, cerebellum

## Abstract

The beneficial effects of *Prosopis africana* (PA) on human health have been demonstrated; however, its protective effects against heavy metals (HM) are not yet understood. This study evaluated the potential neuroprotective effects of PA in the cerebral cortex and cerebellum. To accomplish this, we divided 35 albino Sprague Dawley rats into five groups. Group I did not receive either heavy metal mixture (HMM) or PA. Group II received a HMM of PbCl_2_ (20 mg/kg), CdCl_2_ (1.61 mg/kg), HgCl_2_ (0.40 mg/kg), and NaAsO_3_ (10 mg/kg) orally for a period of two months. Groups III, IV, and V received HMM along with PA at doses of 500, 1000, and 1500 mg/kg, respectively. PA caused decreased levels of HM accumulation in the cerebral cortex and cerebellum and improved performance in the Barnes maze and rotarod tests. PA significantly reduced levels of IL-6 and TNF-α. PA increased concentrations of SOD, CAT, GSH, and Hmox-1 and decreased the activities of AChE and Nrf2. In addition, levels of MDA and NO decreased in groups III, IV, and V, along with an increase in the number of live neurons. In conclusion, PA demonstrates a complex neuroprotective effect with the potential to alleviate various aspects of HM-induced neurotoxicity.

## 1. Introduction

Motor and cognitive deficits, along with psychiatric and behavioral disturbances, are among neurophysiological disruptions caused by neurotoxicants [1],[2]. Humans have been exposed to various environmental toxicants, particularly metals and metalloids, since ancient times, and their toxic effects have been extensively investigated and documented [3]. Despite their unprecedented toxicity to the nervous system even at subliminal concentrations, many countries have implemented regulations to control their levels in different environmental matrices, including food, water, air, soil, etc. [4]. However, despite these measures, long-term exposure to neurotoxic metals through food and water consumption, as well as other routes such as occupational inhalation, tobacco smoking, and, more recently, electronic cigarette vaping [5]–[7], still presents a significant public health challenge.

Unlike other nutrients or essential metals, noxious elements have no known biological benefit to humans. These elements are outrightly noxious and pose risks to human health [8]. In humans, Pb, Hg, Cd, and As are absorbed with varying efficiencies, disrupt the blood-brain barrier, and preferentially accumulate in different regions of the brain where they can potentially cause adverse neurotoxic effects even at low concentrations [9].

Accumulating evidence suggests that there is no safe level of lead exposure [2],[10]. Pb exposure occurs through inhalation of lead-contaminated dust particles, consumption of food and water contaminated with lead, and exposure to lead-based paints commonly found in older homes. Chronic lead exposure leads to neurotoxicity, characterized by significant neurological deficits, including impairments in intelligence, memory, processing speed, reading and comprehension, visuospatial skills, and motor skills [8],[11]. Regarding Cd, its long half-life and poor elimination result in a tendency to bioaccumulate in peripheral organs such as the kidneys, lungs, and testes, also being able to reach the central nervous system (CNS). Cd-associated neurotoxicity is linked to neurodegenerative diseases, including Parkinson's disease, Alzheimer's disease, Huntington's disease, and multiple sclerosis [12]. Inspiration of contaminated dust, fumes, or mists, as well as ingestion of crops harvested from As-contaminated farmlands, are major sources of human exposure to As. In both humans and animals, As is readily absorbed and tends to bioaccumulate in the brain [13].

Heavy metals, particularly Hg, selectively cause degeneration of the nervous system and are toxic to the cerebral and cerebellar cortex [14]. Previous studies have reported toxicity and tissue bioaccumulation profiles of single heavy metals [15],[16], but human exposure to multiple heavy metals usually occurs concertedly, especially in the coastal regions of Niger Delta Nigeria, where we have reported a cocktail of these metals in vegetables, meat, fish, etc. [17],[18].

Nutraceuticals have demonstrated benefits in the management of non-communicable diseases. Recently, bioactive molecules isolated from plants have been extensively studied for therapeutic research. The benefit of nutraceuticals in preventing metal-induced neurotoxicity has garnered significant support, as various plant extracts have exhibited a range of pharmacological properties [19]. Reports suggest that phytoceuticals and nutraceuticals contain potent ingredients that are used in the management of metal-induced neurodegeneration [20],[21]. These agents have been shown to protect against neuronal injuries, attenuate neurotoxicity, and enhance neuronal survival [22].

The seed of *Prosopis africana* (PA) is rich in carbohydrates, fiber, protein [23], potassium, and magnesium, with significant amounts of essential amino acids [24],[25]. The fermentation of PA, known as *Okpeye*, enhances its nutritional value while reducing antinutritional and toxic factors. The PA is widely used in Africa, particularly in Nigeria, as a condiment, flavor enhancer, micronutrient, and protein supplement [26]. Previous studies from our lab demonstrated a significant antioxidant potential of PA [27]. Only recently, hormesis—a process in which small, nontoxic exposure to stress is used to induce adaptive responses that protect biological systems against subsequently large and potentially lethal stresses of the same, similar, or different nature—has generated a lot of interest. The field of hormetic/adaptive responses activated via natural ingredients (polyphenols, flavonoids, etc.) is emerging as a promising field for new nutritional antioxidant strategies in neurodegeneration in animal studies where the vast majority of observations have revealed biphasic dose–response hormetic effects [28],[29].

Oxidative stress plays a prominent role in the neuropathophysiology of various brain-related dysfunctions, particularly neurodegenerative diseases. The nuclear factor-erythroid 2 related factor 2/antioxidant-responsive element Nrf2-ARE system serves as the primary cellular defense against oxidative stress by regulating the expression of antioxidant molecules and enzymes. Dysregulation of this system and the excessive generation of reactive oxygen species (ROS) can damage crucial cellular components, leading to the loss of structural and functional integrity of neurons [30].

The current study aims to evaluate the influence of an aqueous extract of PA on the Nrf2-ARE system in the cerebral cortex and cerebellum of rats exposed to a mixture of heavy metals.

## 2. Methods

### 2.1. Materials

#### 2.1.1. Prosopis africana

PA pods were harvested from Nsukka, Enugu State, Nigeria (latitude: 6.857816/N 60°51′28.138″; longitude: 7.411943/E 70°24′42.996″), identified by Mr. Ozioko, Department of Botany, University of Nigeria, Nsukka, washed, sun-dried for three days, and blended to a powdery form. Subsequently, 100 g of the powder was mixed with 1000 mL of deionized water and shaken for 48 h [31]. The slurry was sieved and filtered through a Whatman filter paper No.1 after vigorous shaking of the mixture. The extract was then separated and stored in a refrigerator at 4 °C. The crude extract was partially purified by sequential extraction using different solvents with different (increasing) polarity: petroleum ether, chloroform, ethyl acetate, and finally methanol.

Soxhlet apparatus (temperature 40 °C, time 2–3 h) was used for the fractionation. This was done by tying the dried methanol extract with a muslin cloth and then inserting into the thimble of the Soxhlet apparatus. Methanol was poured into the distillation flask below and allowed to heat at 40 °C. The vapor resulting from the distillation flask condensed in the thimble holder and dissolved the dried crude extract tied inside the thimble. When the condensed methanol in the extraction chamber reached the overflow level, the solution in the thimble holder was aspirated by a siphon and returned to the distillation flask. This was repeated until all soluble compounds in the methanol were extracted. The sample (dried crude extract) was then removed, untied, and air-dried. The methanol extract was concentrated with the rotary evaporator, and the dried residues were subjected to quantitative phytochemical screening. The methanol fraction was subjected to GC–MS analysis.

#### 2.1.2. PA preparation for gas chromatography–mass spectrometry (GC–MS) analysis

Phytochemical analysis of methanol extracts of PA was carried out using Shimadzu GC–MS QP2010 Plus. At the start, the column oven temperature was set at 70 °C and kept for 5 min; thereafter, it was raised to 250 °C at 10 °C/min and held for 10 min. The temperature was subsequently increased to 300 °C at intervals of 10 °C per minute and held for another 10 min. The instrument specifications were as follows: pressure, 110.8 kPa; injection mode, splitless; total flow, 38.9 mL/min; column flow, 1.71 mL/min; solvent cut time, 3.5 min; detector gain mode, relative; injection temperature, 280 °C; purge flow, 3 mL/min; and sample injection volume, 2 µL. The detector operated at a temperature of 320 °C and helium was the carrier gas. Compounds were identified in the samples by comparing the mass spectra with the National Institute of Standards and Technology and Wiley 7 library.

### 2.2. Experiments

A total of 35 male albino Sprague Dawley rats (n = 35; 90 days old) were sourced from the Department of Toxicology, University of Port Harcourt, Nigeria Animal House. The rats were placed in clean cages with two rats per cage and allowed to acclimate for 14 days under a 12-h reverse light/dark cycle (lights on at 7:00 a.m.). Throughout the acclimatization period and subsequent experimental phases, the rats had access to both feed and water. All experimental procedures were conducted in accordance with the guidelines outlined by the Animal Use and Care Committee at the University of Port Harcourt, Nigeria (UPH/CEREMAD/REC/MM73/014) and were consistent with the principles established in the Guide for the Care and Use of Laboratory Animals by the National Institutes of Health.

For a duration of 60 days, weight-matched Sprague Dawley male albino rats (7 rats per group) were assigned at random to five groups subjected to different treatments. The control group (group 1) received deionized water exclusively; group 2 was administered environmentally relevant doses of a mixture of heavy metals, namely PbCl_2_ (20 mg/kg), CdCl_2_ (1.61 mg/kg), HgCl_2_ (0.40 mg/kg), and NaAsO_3_ (10 mg/kg), as specified by [32] and [33]. Groups 3, 4, and 5 were also exposed to the metal mixture plus PA at doses of 500 mg/kg, 1000 mg/kg, and 1500 mg/kg, respectively [31]. There were no observable side effects of PA at the doses applied in this study. All treatments were administered through oral gavage for 60 days. The ARRIVE checklist was used to standardize the protocol and ensure transparency.

### 2.3. Behavioral tests

Each rat was brought from the holding room to the testing room immediately prior to testing. Rats were tested in both the morning (9:00 a.m. to 12:00 p.m.) and afternoon (1:00 p.m. to 4:00 p.m.) in the Barnes maze and rotarod tests. Rats in the rotarod test were tested a total of six times (three times a day over two days); rats in the Barnes maze test were not tested more than once [34]. Behavioral tests were carried out from day 50.

#### 2.3.1. Barnes maze testing

The Barnes maze is a circular white Plexiglas platform (160 cm in diameter) with 18 circular holes and surrounded by a 45 cm high wall [35]. Holes were blocked with mesh but the target hole was left unblocked. A plastic transparent escape cage was placed under the escape hole, filled with bedding from the home cage. The start box was a white, open-ended cylinder, easily lifted from the platform to the roof, approximately 3 m above. The maze was positioned in a room with many extra-maze cues to allow the orientation of rats in the space.

The Barnes maze platform was evenly illuminated (300 lx) by fluorescent lights located on the ceiling. The procedure consisted of habituation trial, acquisition trials, and retention trial [35]. The habituation session started by putting the rats into the transparent escape box (filled with bedding from the home cage) for 120 s. Afterward, the rat was put near the escape hole surrounded by the start box and given 60 s to escape. If the rat did not enter the escape box within that time, it was carefully lifted and guided through the target hole into the escape box. The rat was left in the escape box for 120 s. Finally, the rat was put in the center of the maze and left undisturbed for the following 240 s for it to enter the escape box. If the rat failed to enter the escape box during the assigned time, it was placed into the escape box manually following the aforementioned procedure and kept there for 120 s.

Habituation sessions were separated by a 300-s resting period. In this resting period, the rat remained in its home cage [36]. After two days of habituation bout, rats were trained for five days and thereafter exposed to a retrieval stretch seven days later. Each training or retrieval bout comprised of four consecutive 270-s trials, separated by a 300-s resting period in the rat home cage. At the onset of each trial, each rat was kept in the start box in the center of the maze for 30 s until the trial was commenced by lifting the cylinder; subsequent free maze exploration occurred for the next 240 s. If the rat failed to enter the escape box within the assigned time, it was gently lifted and put in the escape box. The rat was left in the escape box for 120 s, after which it was returned to its home cage for 300 s. The escape hole remained at a constant position throughout all trials and sessions.

The time for locating the target hole was taken as the escape latency and recorded. All behavioral tests were blinded to the treatment groups to minimize bias.

#### 2.3.2. Rotarod test

Rats were trained to walk on the accelerating rotarod (UgoBasile 47600, Milan, Italy). The rotarod comprised a cylinder with a diameter of 3 cm on which five rats could run simultaneously, separated by panels of sufficient size to prevent them from detecting each other visually. The speed of the rod was linearly increased from 4 rpm to 40 rpm for 300 s, after which the rats were returned to their cages. Once the rat was incapable of maintaining its balance and fell off the device, a sensor was triggered, and the time was recorded. The first week of the experiment was used to train the rats in the use of the device and to obtain baseline values. Rats were subjected to the rotarod on a daily basis [37],[38].

### 2.4. Necropsy, harvesting cerebral cortex and cerebellum, and ELISA assays

Rats were euthanized using pentobarbital anesthesia (IP 50 mg/kg). The brain from each rat was harvested with meticulous isolation of the cerebrum and cerebellar regions. Each of these regions was divided into two portions and stored at −80 °C. One portion was for metal analysis and the other half was used for biochemical analysis.

The cerebral cortex and cerebellum were homogenized separately in 9 volumes of cold phosphate buffer (0.1 M, pH 7.4). The homogenates were then subjected to centrifugation at 3000 rpm for 20 min at 4 °C to remove any nuclear debris. The cerebral cortex and cerebellum lysates were used for the assessments of acetylcholinesterase (AChE), MDA, NO, GSH, GPx, GST, and SOD, as well as ELISA assays targeting TNF-α, IL-6, NF-kB, Nrf2, hmox-1, and Casp-3 previously described in our lab according to manufacturer's instructions [39],[40].

### 2.5. Antioxidant and oxidative stress markers in the cerebral cortex and cerebellum

Glutathione peroxidase (GPx) activity was measured using the method described by Paglia and Valentine (1967) [41], with absorbance measured at 412 nm. Reduced glutathione (GSH) levels were determined following the procedure outlined by Jollow et al. (1974) [42], with absorbance measured at 412 nm. Glutathione S-transferase (GST) activity was determined using the method of Habig et al. [43], with absorbance measured at 310 nm. Superoxide dismutase (SOD) activity was assessed separately in the cerebral cortex and cerebellum [44]. Catalase (CAT) activity was determined by monitoring the breakdown of H_2_O_2_ at 240 nm, following Aebi's method [45]. Lipid peroxidation, evaluated as thiobarbituric acid reactive substances (TBARS), was determined by the adaptation of Esterbauer and Cheeseman's method (1990) [46]. Nitric oxide (NO) was assayed using the Griess reaction technique [47], with absorbance measured at 540 nm against the blank.

### 2.6. Determination of heavy metals

The harvested cerebral cortex and cerebellum were treated as previously described by Ikpeama et al. 2023 [48] and Pb, Cd, and As were determined with an atomic absorption spectrometer [49].

### 2.7. Histopathology

After the behavioral test, two rats per group were euthanized with 50 mg/kg pentobarbital IP and perfused with heparinized saline 0.9% solution followed by 4% paraformaldehyde in 0.2 M phosphate buffer. The histopathology of the cerebral cortex and cerebellum was done by a histopathologist who was blinded to the experimental groups. The number of live cells in the cerebral cortex and cerebellum of each group was also quantified with ImageJ version 1.48 software using standard Hounsfield unit ranges by delineating regions of interest (ROI) with a mouse computer interface by using the region selection tools in Image J Menu toolbar.

### 2.8. Molecular docking

Since the perceived protection from HMM is through a PA effect on acetylcholine esterase (AChE), AChE docking was included. The crystallized structure of AChE with ID: 3M3D was downloaded from the Protein Data Bank. The protein was prepared prior to molecular docking using AutoDock tools; this involved the removal of co-crystallized ligand, addition of charges, polar hydrogen, and grid setup. The 3D structure of resveratrol and galantamine (an FDA-approved AChE inhibitor) was downloaded from Pubchem (SDF file). These SDF files were then converted to PDB files using Pymol software [50]. However, the 3D structure of Model Oris (the modeled compound) was generated and optimized using Molecular Operating Environment (MOE) package (The MOE, 2015), and converted to PDB using Chem 3D software. Molecular docking of the ligand against AChE (target protein) was performed using AutoDock Vina software [51], and the binding affinities/energy were reported in kcal/mol. Resveratrol modeling by scalphold replacement was carried out using MOE package. This involved replacing the non-polar alkene groups linking the two polar ends of resvestarol, thereby optimizing this portion for further interaction within AChE, and hence improving its physiological roles. The model was further docked with the same grid option as in resveratrol and galantamine and the docking scores were reported.

### 2.9. Statistical analysis

Data were expressed as mean ± standard deviation (SD), with n = 5 except for behavioral studies, where n = 7. Microsoft Xlstat 2014 was used to perform analysis of variance and Tukey's multiple-comparison pairwise tests to check if the concentration of the biomarkers was significantly different between groups. The data analysis involved performing descriptive statistics of the metals and biomarkers concentration before ANOVA was used to establish if there was a significant difference in the concentration of the heavy metals and biomarkers among groups. All significant differences were at a p < 0.05.

## 3. Results

### 3.1. Phytoconstituents identified by GC–MS in aqueous extract PA

Table 1 presents the phytoconstituents detected in the water extract of PA; 40% of the constituents are flavonoids, 27% are alkaloids, 13% are polyphenols, 12% are phenolic acids, and 5% are amino acids.

**Table 1.** Phytoconstituents detected in aqueous extract of *Prosopis africana* (PA) with chemical characteristics, concentration, and relevant activity.

No	R. Time (minutes)	Molecular formula	Molecular weight g/mol	Compound name	Compound class	Concentration (ug/g)	Activity
1	8.91	C_20_H_28_O_5_	348.40	Cuhumulone or Cohumulone	Phenolic acid	3.355	Antioxidant, anti-inflammatory
2	10.65	C_21_H_30_O_5_	362.50	Humulone	Phenolic acid	3.786	Antioxidant, anti-inflammatory
3	12.27	C_22_H_32_O_5_	376.50	Adhumulone	Phenolic acid	4.470	Antioxidant, anti-inflammatory
4	13.74	C_15_H_12_O_2_	224.25	Flavonones or flavanones	Flavonoids	4.143	Antioxidant, anti-inflammatory
5	14.69	C_6_H_13_N_3_O_3_	175.19	Citrulline	Amino acid	4.572	Improve SOD, CAT, GPx, and NO
6	16.04	C_14_H_12_O_3_	228.24	Resveratrol	Polyphenol	4.250	Antioxidant, anti-inflammatory
7	17.09	C_15_H_14_O_6_	290.27	Catechin	Polyphenol	4.100	Antioxidant, anti-inflammatory
8	18.05	C_15_H_26_N_2_	234.38	Sparteine	Alkaloid	7.723	Bivalent chelator
9	19.52	C_15_H_17_NO_4_	275.30	Ribalindine	Alkaloid	11.149	ROS scavenging
10	20.59	C_15_H_14_O_2_	226.27	Flavan-3-ol	Flavonoids	6.315	Antioxidant
11	21.67	C_15_H_14_O_6_	290.27	Epicatechin	Polyphenol	1.372	Antioxidant
12	22.60	C_27_H_32_O_14_	580.50	Naringin	Flavonoid	14.462	Antioxidant, anti-inflammatory
13	23.23	C_15_H_12_O_5_	272.25	Naringenin	Flavonoid	5.156	Antioxidant, anti-inflammatory
14	23.97	C_31_H_28_O_12_	592.50	Proanthocyanin	Polyphenol	2.887	Antioxidant
15	25.09	C_15_H_10_O_2_	222.24	Flavone	Flavonoids	4.977	Antioxidant
16	25.62	C_12_H_20_N_2_O	208.30	Ammodendrine	Alkaloid	3.299	-
17	26.24	C_15_H_22_N_2_O	246.35	Aphyllidine	Alkaloid	1.037	-
18	26.99	C_11_H_16_N_2_O	192.26	Dohydrocystisine	-	1.286	-

The effect of PA on the body weight and absolute and relative weight of the cerebral cortex and the cerebellum of male albino rats treated with HMM is shown in Table 2. There were no significant differences in the absolute and relative weights of the cerebral cortex and cerebellum between the control and HMM-only exposed rats. Similarly, there were no significant differences in the absolute and relative weights of the cerebral cortex and cerebellum between the HMM-only exposed rats and the HMM-plus-PA co-treated groups. Rats in the different groups showed an increase in body weight, feed intake, and fluid intake.

**Table 2.** Effect of *Prosopis africana* (PA) on the body weight and absolute and relative weight of cerebral cortex and cerebellum of male albino rats treated with heavy metal mixture (HMM).

**Treatment**	**Cerebral cortex**		**Cerebellum**				
	*Absolute (g)	*Relative (%)	*Absolute (g)	*Relative (%)	**Body weight (g)**	**Feed intake**	**Fluid intake**
Deionized H_2_O (only)	0.91±0.18^a^	0.34±1.30	0.30±0.04^a^	0.11±0.29	I = 175±4.36^a^	164.75±18.80^a^	225.08±58.95^a^
					F = 271.33±13.8^a^
					%diff. = 55.05
HMM (only)	0.82±0.10^a^	0.34±0.60	0.28±0.07^a^	0.12±0.42	I = 158±2^b^	78.78±27.54^c^	102.30±20.03^d^
					F = 240±16.64^ab^
					%diff. = 51.90		
HMM + 500 mg/kg PA	0.83±0.03^a^	0.36±0.21	0.30±0.01^a^	0.13±0.07	I = 151±1^c^	88.53±20.90^c^	148.10±27.13^c^
					F = 230.33±14.57^ab^
					%diff. = 52.54		
HMM + 1000 mg/kg PA	0.82±0.05^a^	0.38±0.18	0.27±0.06^a^	0.13±0.22	I = 146±1^cd^	130.03±18.48^b^	190.20±56.50^b^
					F = 213.33±27.15^b^
					%diff. = 46.12		
HMM + 1500 mg/kg PA	0.78±0.04^a^	0.37±0.19	0.24±0.02^a^	0.12±0.10	I = 141±1.73^d^	158.55±18.4^a^	218.31±58.98^a^
					F = 208.33±20.82^b^
					%diff. = 47.75		

*Values = mean ± SD, n = 5, data with different superscripts (a, b, c) are significantly different from each other (*p* < 0.05), data with the same superscripts are not significantly different; HMM: heavy metal mixture. *I = Initial weight, F = Final weight, %diff. = % difference.

Tables 3a and 3b illustrate the effect of PA on the bioaccumulation of As, Pb, and Cd (mg/kg) in the cerebral cortex and cerebellum, respectively, of rats exposed to a heavy metal mixture. There was a significant bioaccumulation of As, Pb, and Cd in the cerebral cortex and cerebellum of HMM-exposed animals. The aqueous extract of PA caused a significant (*p* < 0.05) decrease in the levels of As, Pb, and Cd in the cerebral cortex and cerebellum of rats.

**Table 3a.** Effect of *Prosopis africana* (PA) on the bioaccumulation of As, Pb, and Cd (mg/kg) in the cerebral cortex of rats exposed to heavy metal mixture (HMM).

**Treatment**	**Arsenic**	**Lead**	**Cadmium**
	Mean ± SD	Mean ± SD	Mean ± SD
Control	0.01±0.01^a^	0.02±0.01^a^	0.01±0.01^a^
HMM	1.47±0.14^b^	3.24±0.16^b^	2.75±0.05^b^
HMM + 500 mg/kg PA	0.77±0.11^c^	2.33±0.19^c^	1.97±0.09^c^
HMM + 1000 mg/kg PA	0.76±0.07^c^	2.29±0.11^c^	1.06±0.05^c^
HMM + 1500 mg/kg PA	0.14±0.21^a^	1.03±0.05^a^	0.20±0.03^a^

*Values = mean ± SD, n = 5, data with different superscripts (a, b, c) are significantly different from each other (*p* < 0.05), data with the same superscripts are not significantly different; HMM: heavy metal mixture.

**Table 3b.** Effect of *Prosopis africana* (PA) on the bioaccumulation of As, Pb, and Cd (mg/kg) in the cerebellum of rats exposed to heavy metal mixture (HMM).

Treatment	Arsenic	Lead	Cadmium
	Mean ± SD	Mean ± SD	Mean ± SD
Control	0.03±0.01^a^	0.03±0.01^a^	0.03±0.01^a^
HMM	1.11±0.15^b^	4.30±0.16^b^	2.75±0.053^b^
HMM + 500 mg/kg PA	0.96±0.14^b^	2.03±0.17^c^	2.51±0.32^b^
HMM + 1000 mg/kg PA	0.46±0.11^c^	1.90±0.03^c^	1.22±0.07^c^
HMM + 1500 mg/kg PA	0.10±0.08^a^	0.61±0.09^a^	0.36±0.11^a^

*Values = mean ± SD, n = 5, data with different superscripts (a, b, c) are significantly different from each other (*p* < 0.05), data with the same superscripts are not significantly different; HMM: heavy metal mixture.

The performance of rats treated with PA in the rotarod test is presented in Table 4. The control group exhibited a significantly higher latency of fall from the rotating wheel/rod of the rotarod than all the other groups (p < 0.05, df = 4, F = 2.4). The group treated with only the heavy metal mixture showed a significantly lower latency of fall, indicating neuromuscular coordination and balance abnormalities. The groups co-treated with PA had significantly higher latencies of fall compared to the HMM group.

**Table 4.** Effect of *Prosopis africana* (PA) on the rotarod and Barnes maze tests (time in seconds).

**Groups**	**Rotarod test**	**Barnes maze test**
Deionized water	11.00±3.53^a^	20.39±2.95^a^
Heavy metals	6.33±1.01^b^	61.29±4.16^b^
HMM + 500 mg/kg PA	7.50±0.33^a^	49.44±6.73^c^
HMM + 1000 mg/kg PA	8.22±0.63^b^	30.11±8.25^d^
HMM + 1500 mg/kg PA	10.78±3.40^c^	25.95±4.01^d^

Note: Values are expressed as mean ± SD, n = 7. Different superscripts (a, b, c, d) indicate a statistically significant difference between the means at *p* < 0.05, while similar superscript letters have no significant difference.

Table 4 also displays the performance of rats treated with PA in the Barnes maze test. The control group had a significantly lower escape latency in locating the hole compared to the group exposed only to the heavy metal mixture (p < 0.05, df = 4, F = 8.81). The groups treated with PA had significantly lower escape latencies compared to the HMM-only group.

The effect of PA on the levels of MDA (µmol/mL) and NO (µM/L) in the cerebral cortex and cerebellum of male albino rats treated with HMM is shown in Table 5. Treatment with the HMM significantly increased both MDA (µmol/mL) and NO (µM/L) levels compared with the control group that received deionized water (*p* < 0.05). Administration of aqueous PA significantly reduced the concentrations of both MDA (µmol/mL) and NO (µM/L) compared with the rats exposed to HMM alone.

**Table 5.** Effect of *Prosopis Africana* (PA) on the MDA (µmol/mL) and NO (µM/L) levels of heavy metal mixtures (HMM)-treated cerebral cortex and cerebellum of male albino rats.

Treatment	**MDA**		**NO**	
	**CC**	**CE**	**CC**	**CE**
Control	0.51±0.10^a^	0.41±0.13^a^	6.55±0.65^a^	4.85±1.46^a^
HMM	0.78±0.03^b^	0.75±0.06^b^	13.53±3.12^b^	14.33±6.93^b^
HMM + 500 mg/kg PA	0.39±0.10^c^	0.46±0.13^a^	4.53±1.60^c^	3.22±1.00^a^
HMM + 1000 mg/kg PA	0.50±0.16^a^	0.48±0.13^a^	7.00±3.30^a^	4.49±1.13^a^
HMM + 1500 mg/kg PA	0.32±0.22^c^	0.46±0.01^a^	9.87±3.93^d^	6.23±0.83^c^

*Values = mean ± SD, n = 5, data with different superscripts (a, b, c) are significantly different from each other (*p* < 0.05), data with the same superscripts are not significantly different; HMM: heavy metal mixture.

There was a significant decrease (*p* < 0.05) in the antioxidants SOD, CAT, and GSH in the cerebral cortex and cerebellum of rats exposed only to HMM compared with the control group that received deionized water (Table 6). However, co-treatment with the aqueous extract of PA and HMM caused a significant increase (*p* < 0.05) in SOD, CAT, and GSH levels in the cerebral cortex and cerebellum. Co-treatment with the aqueous extract of PA and HMM did not result in a significant decrease in GPx levels in the cerebral cortex and cerebellum (Table 6).

**Table 6.** Effect of *Prosopis africana* (PA) on antioxidants [SOD (U/mg), GPx (IU), CAT (mM), GSH (µmol/mL)] in the cerebral cortex and cerebellum of HMM-treated male albino rats.

**Treatment**	**SOD**		**GPx**		**CAT**		**GSH**	
	**CC**	**CE**	**CC**	**CE**	**CC**	**CE**	**CC**	**CE**
Control	0.30±0.10^a^	0.28±0.02^a^	0.11±0.01^a^	0.09±0.01^a^	2.32±0.24^a^	1.24±0.99^a^	1.17±0.23^a^	1.43±0.48^a^
HMM	0.12±0.01^b^	0.13±0.03^b^	0.08±0.01^a^	0.07±0.01^a^	0.68±0.21^b^	0.76±0.24^b^	0.91±0.12^b^	0.64±0.10^b^
HMM + 500 mg/kg PA	0.42±0.14^c^	0.33±0.12^a^	0.08±0.01^a^	0.07±0.01^a^	3.42±0.15^c^	4.13±1.06^c^	1.15±0.07^a^	1.05±0.09^a^
HMM + 1000 mg/kg PA	0.35±0.11^a^	0.35±0.05^a^	0.09±0.02^a^	0.09±0.01^a^	3.99±0.17^c^	3.95±0.86^c^	1.23±0.22^a^	1.36±0.26^a^
HMM + 1500 mg/kg PA	0.50±0.15^b^	0.37±0.12^c^	0.09±0.01^a^	0.08±0.01^a^	3.96±0.76^c^	3.41±1.23^c^	1.40±0.18^c^	1.15±0.08^a^

*Values = mean ± SD, n = 5, data with different superscripts (a, b, c) are significantly different from each other (*p* < 0.05), data with the same superscripts are not significantly different; HMM: heavy metal mixture.

Table 7 illustrates the effect of PA on the pro-inflammatory cytokines (pg/mL) in the cerebral cortex and cerebellum of male albino rats treated with a heavy metal mixture. Treatment with the heavy metal mixture alone caused a significant increase (*p* < 0.05) in IL-6 and TNF-α levels compared with the control group. However, administration of the aqueous extract of PA resulted in a dose-dependent and significant decrease (*p* < 0.05) in IL-6 and TNF-α levels compared with rats exposed to the HMM alone.

**Table 7.** Effect of *Prosopis africana* (PA) on the pro-inflammatory cytokines (pg/mL) in the cerebral cortex and cerebellum of HMM-treated male albino rats.

**Treatment**	**IL-6**		**TNF-α**	
	**CC**	**CE**	**CC**	**CE**
Control	41.90±8.48^a^	23.63±6.30^a^	34.53±7.4^a^	32.40±5.82^a^
HMM	68.03±8.72^b^	50.70±7.83^b^	68.03±6.56^b^	56.53±8.89^b^
HMM + 500 mg/kg PA	67.67±8.82^b^	57.43±5.91^c^	34.80±6.13^a^	48.13±5.62^c^
HMM + 1000 mg/kg PA	54.23±4.67^c^	53.33±7.42^c^	44.10±4.10^c^	42.00±2.29^c^
HMM + 1500 mg/kg PA	31.53±4.24^d^	44.73±8.10^d^	30.93±7.5^d^	17.40±6.02^d^

*Values = mean ± SD, n = 5, data with different superscripts (a, b, c, d) are significantly different from each other (*p* < 0.05), data with the same superscripts are not significantly different; HMM: heavy metal mixture.

The effect of aqueous PA on the levels of Nrf2 (pg/mL), Nf-kb (pg/mL), and Hmox-1 (pg/mL) in the cerebral cortex and cerebellum of male albino rats treated with the heavy metal mixture is presented in Table 8. Treatment with the heavy metal mixture alone caused a significant increase (*p* < 0.05) in Nrf2 levels, a significant decrease (*p* < 0.05) in Hmox-1 levels, and a non-significant increase in Nf-kb levels. Co-treatment with HMM + PA at doses of 500, 1000, and 1500 mg/kg significantly decreased Nrf2 levels in the cerebral cortex, whereas co-treatment with HMM + PA at doses of 1000 and 1500 mg/kg significantly decreased Nrf2 levels in the cerebellum compared to the HMM-only group. There was a significant increase (*p* < 0.05) in Hmox-1 levels after co-administration of HMM + PA.

**Table 8.** Effect of *Prosopis africana* (PA) on the Nrf2 (pg/mL), Nfkb (pg/mL), and Hmox-1 (pg/mL) in the cerebral cortex and cerebellum of HMM-treated male albino rats.

**Treatment**	**Nrf2**		**NF-Kb**		**Hmoxl**	
	**CC**	**CE**	**CC**	**CE**	**CC**	**CE**
Control	7.27±0.29^a^	6.53±0.99^a^	0.07±0.01^a^	0.08±0.00^a^	1.24±0.13^c^	0.93±0.64^a^
HMM	12.03±1.63^b^	11.93±1.06^b^	0.09±0.01^a^	0.09±0.02^a^	0.57±0.37^b^	0.65±0.05^b^
HMM + 500 mg/kg PA	10.77±0.59^b^	11.27±1.32^b^	0.12±0.01^a^	0.12±0.03^a^	0.85±0.30^c^	0.67±0.03^a^
HMM + 1000 mg/kg PA	8.90±0.17^c^	7.50±0.95^c^	0.09±0.01^a^	0.09±0.03^a^	0.99±0.36^c^	0.70±0.18^a^
HMM + 1500 mg/kg PA	7.83±0.58^c^	7.80±0.36^c^	0.11±0.03^a^	0.10±0.00^a^	1.07±0.20^c^	0.70±0.18^a^

*Values = mean ± SD, n = 5, data with different superscripts (a, b, c) are significantly different from each other (*p* < 0.05), data with the same superscripts are not significantly different; HMM: heavy metal mixture.

The effect of the aqueous extract of PA on caspase 3 levels in the cerebral cortex and cerebellum of male albino rats treated with a heavy metal mixture is shown in Table 9. Treatment with the heavy metal mixture alone caused a significant increase (*p* < 0.05) in the level of caspase 3 in the cerebellum compared to the control group. Co-treatment of HMM with the aqueous extract of PA resulted in a significant decrease (*p* < 0.05) in the level of caspase 3 compared to the HMM-only treated group. However, the administration of the aqueous extract of PA did not show a significant (*p* > 0.05) reduction in the level of caspase 3 in the cerebral cortex of HMM-exposed rats.

**Table 9.** Effect of *Prosopis africana* (PA) on Caspase 3 (µmol/mL) in the cerebral cortex and cerebellum of HMM-treated male albino rats.

**Treatment**	**Caspase 3**	
	**CC**	**CE**
Control	0.52±0.16^a^	0.21±0.07^a^
HMM	0.58±0.17^a^	0.64±0.16^b^
HMM + 500 mg/kg PA	0.59±0.21^a^	0.50±0.02^c^
HMM + 1000 mg/kg PA	0.55±0.18^a^	0.52±0.12^c^
HMM + 1500 mg/kg PA	0.50±0.27^a^	0.44±0.08^c^

*Values = mean ± SD, n = 5, data with different superscripts (a, b, c) are significantly different from each other (*p* < 0.05), data with the same superscripts are not significantly different; HMM: heavy metal mixture.

#### 3.1.1. Molecular docking

**Figure 1.**
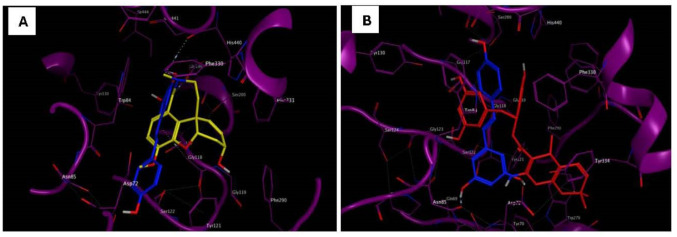
(a) Superimposed view of the active-site interaction of resveratrol (blue color) and galantamine (in yellow). (b) Superimposed view of the active-site interaction of resveratrol (blue color) and Model Oris (in red).

Several inhibitors of AChE are composed mainly of two pharmacophores joined via an appropriate group or chain. During catalysis, these pharmacophores/polar groups bind simultaneously to the catalytic and peripheral sites of AChE, which are approximately 14 Å from each other, found at the top and the bottom of the AChE gorge (Figure 1). Resveratrol presented, in the structure displays, various forms of interactions (hydrophobic) with a binding affinity of −9.0 kcal/mol, which is responsible for its AChE inhibitory activity. The molecular docking study showed that the hydroxyl group attached to the benzene ring of resveratrol present at both ends formed a hydrogen bond with the carboxylic group of Glu199 in the peripheral pocket and the OH group at the backbone of Tyr70 at the other end, enabling the strengthening of its binding affinity; the rest of resveratrol formed hydrophobic interactions with Ser122, Tyr121, Phe330, and Trp84. Galantamine (standard drug) was also found to be interacting with similar residues at the active site, though with a lesser binding affinity of −8.9 kcal/mol, implying the therapeutic potential of resveratrol as a AChE inhibitor.

**Figure 2.**
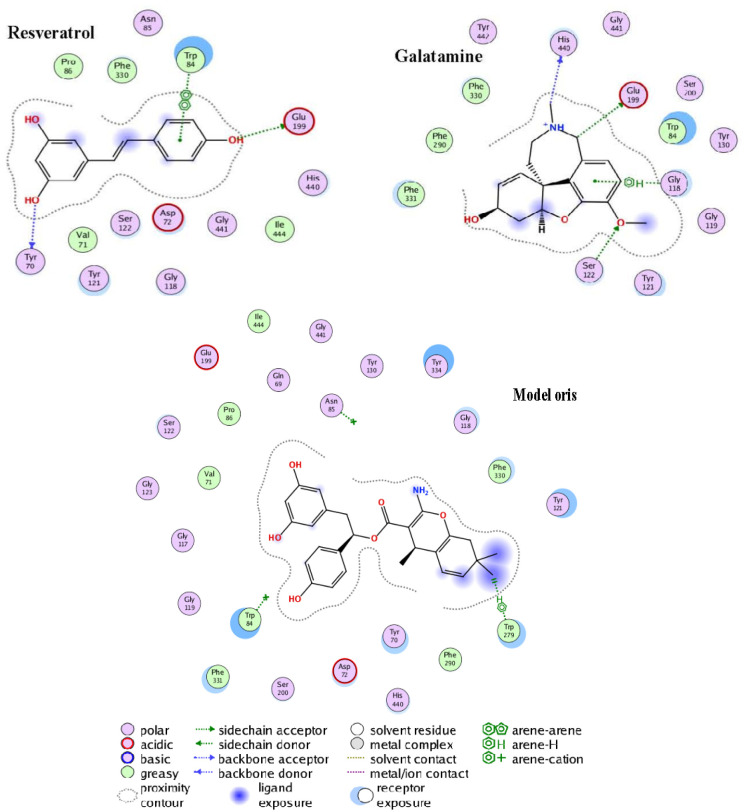
Acetylcholine esterase (AChE) active site interactions (2D diagram) docked compounds showing amino acid residues forming major interactions. Hydrogen-bonding interactions of ligands and residues are shown in a green line. More details of the interactions are shown in the attached legend.

However, an active-site view of the resveratrol–AChE complex revealed possible points of optimization in resveratrol in order to boost its therapeutic benefits (Figure 2). Due to the non-polar alkene group joining both reactive ends of resveratrol, there was no interaction at this region. A fragment modeling through scaffold replacement of these groups was performed using MOE, 2015 while placing a pharmacophore filter to keep key interactions prior to scalphold replacement search. Over 22,856 fragments of replaceable linkers (scaffolds) were generated, from which the best was picked (Model Oris). Molecular docking study of Model Oris against AChE gave a binding affinity of −10.6 kcal/mol, much higher than resveratrol and galantamine. This could be because of more interactions initiated at AChE active sites (Try334, Gln69, His440, Try84, and Ser200) following resveratrol modeling.

Table 10 presents the effect of the aqueous extract of PA on AChE activity in the cerebral cortex and cerebellum of male albino rats treated with a heavy metal mixture. Administration of the heavy metal mixture alone showed a significant increase (*p* < 0.05) in AChE activity in the cerebral cortex (237.40 ± 159.35) and cerebellum (294.30 ± 135.71) compared to the control group (32.40 ± 3.24 and 66.67 ± 14.44 in the cerebral cortex and cerebellum, respectively). Co-treatment of HMM with the aqueous extract of PA caused a significant reduction in AChE activity in both the cerebral cortex and cerebellum compared to the HMM-only treated group.

**Table 10.** Effect of *Prosopis africana* (PA) on the AChE (µmol/mL) activity in the cerebral cortex and cerebellum of HMM-treated male albino rats.

**Treatment**	**AChE**	
	**CC**	**CE**
Control	32.40±3.24^a^	66.67±14.44^a^
HMM	237.40±159.35^b^	294.30±135.71^b^
HMM + 500 mg/kg PA	130.23±175.89^c^	85.90±8.00^d^
HMM + 1000 mg/kg PA	119.50±104.55^c^	101.13±17.75^c^
HMM + 1500 mg/kg PA	82.53±47.91^d^	104.27±40.64^c^

*Values = mean ± SD, n = 5, data with different superscripts (a, b, c) are significantly different from each other (*p* < 0.05), data with the same superscripts are not significantly different; HMM: heavy metal mixture.

### 3.2. Histopathology

**Figure 3.**
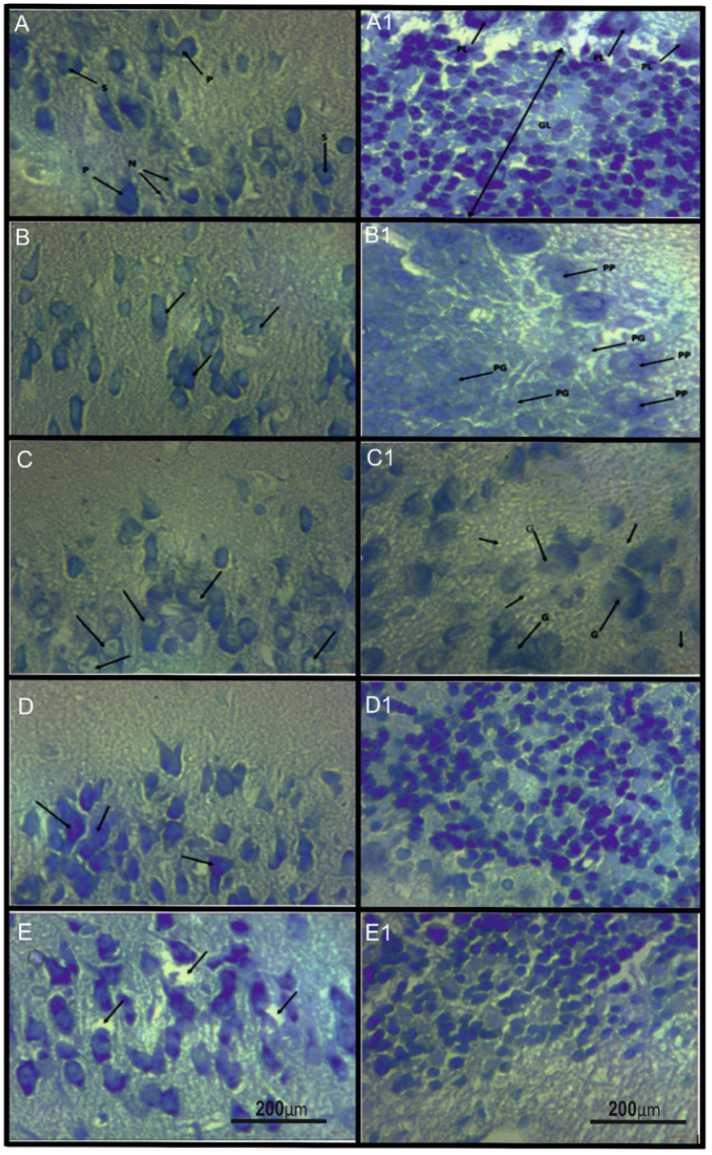
Histopathological findings in five examined groups of animals (A–E: cerebral cortex; A1–E1: cerebellum).

Photomicrographs (×400, toluidine blue) (Figure 3A–E) of the cerebral cortex are shown. The first image displays normal neuronal cells, indicating their healthy state. In the second image, cell loss is observed in the outer granular layer, accompanied by abnormal neuronal cell morphology (arrow). The third image reveals cell loss and diffuse neuronal cell cytoplasmic vacuolation. The fourth image displays the outer granular layer containing numerous normal neuronal cells (arrows). Finally, the fifth image (GRP 5 HMM + 1500 mg/kg PA CC ×400) exhibits restored neurons with minimal neutrophil vacuolation (arrows) in the outer granular layer.

In Figures 3A1–E1, photomicrographs (×400, toluidine blue) of the cerebellum are presented. The first image depicts the normal cerebellar architecture, showcasing the Purkinje cell (PL) and granule cells in the granular layer. In the second image, multiple pyknotic Purkinje cells (PP) and granule cells (PG) are observed. The third image reveals moderate cell loss with hypertrophy of scanty cells (G) and diffuse neutrophil vacuolation (arrows). The fourth image displays a restored density of granular cells in the cerebellum. Lastly, the fifth image exhibits restored cells in the cerebellum.

## 4. Discussion and conclusion

This study evaluated the behavioral changes induced by exposure to a quaternary metal mixture in rats, as well as the potential protective effects of the aqueous extract from *Prosopis africana* (PA) seeds. The findings confirm that chronic exposure to the quaternary metal mixture has a negative impact on memory retention and balance, as assessed by the Barnes maze test and the rotarod test, respectively. These effects may be attributed to the ability of the metal mixture to interfere with downstream effector molecules that are essential for long-term potentiation. Impairment of these molecules could lead to memory loss and neurobehavioral changes [52],[53]. The parasympathetic neurotransmitter acetylcholine is crucial for learning and memory [54]. The neurotransmitter acetylcholine, which plays a crucial role in learning and memory [54], is affected by the metal mixture. The primary degradation enzyme of acetylcholine, acetylcholinesterase (AChE), is widely recognized as an indicator of damage to cholinergic neurons in the brain cleft [55]. Furthermore, lead (Pb) interferes with neurotransmitter release and disrupts the functions of cholinergic systems and gamma-aminobutyric acid (GABA) signaling. It also inhibits N-methyl D-aspartate (NMDA) ion channels [2]. Although no significant differences were observed in the absolute and relative weights of the cerebral cortex and cerebellum between the control group exposed to deionized water and the group exposed only to the quaternary metal mixture, as well as between the group exposed only to the metal mixture and the group co-treated with the metal mixture and PA extract, significant memory and motor impairments were detected in the rats exposed to the metal mixture in the present study. This was evident from the results of the Barnes maze and rotarod performance tests, respectively. Both impairments were significantly reversed by the administration of PA. Moreover, the considerable bioaccumulation of As, Pb, and Cd in the cerebral cortex and cerebellum of the rats exposed to the metal mixture was significantly reduced in a dose-dependent manner by the co-administration of PA extract. Therefore, it is reasonable to suggest that these observations can be attributed, at least in part, to the beneficial effects of PA in reversing the memory and motor impairments caused by the metal mixture.

Oxidative stress plays a significant role in the process of tissue damage. It occurs when an imbalance arises between the production of reactive oxygen species (ROS) and the body's ability to neutralize or repair their harmful effects [56]. Over time, persistent oxidative stress can contribute to different organs toxicity. Managing oxidative stress and bolstering antioxidant defenses are essential strategies in preventing and mitigating tissue damage and its associated health consequences [57].

Some bioactive compounds of PA have been reported to have antioxidant and anti-inflammatory properties [58],[59]. Resveratrol, one of the active ingredients of PA has been demonstrated to inhibit MDA and IL-1β levels, while improving the levels of SOD, CAT, GSH, and GPx [60]. Catechin, a major component of tea, exists in various forms such as epicatechin, epigallocatechin, epicatechin gallate, and epigallocatechin gallate. These forms possess potent antioxidant properties that can effectively neutralize ROS and RNS, thereby contributing to their antioxidant and anti-inflammatory effects [61]. Epicatechins, polyphenolics found in PA, possess both antioxidant and anti-inflammatory properties [62],[63].

Our study demonstrated that exposure to a quaternary metal mixture significantly increased the activity of both cortical and cerebellar acetylcholinesterase (AChE) in rats. This increased AChE activity might be attributed to allosteric interactions between metallic cations and the anionic sites of AChE in the brain [64]. Antioxidant and anti-inflammatory flavonoids and other polyphenols found in PA have shown efficacy in the treatment of Alzheimer's disease by targeting specific enzymes like AChE [65],[66]. The flavonoid quercetin 3-(20,60-diacetylglucoside) has been reported to inhibit AChE activity [67]. Additionally, in another study, the significant reduction in AChE activity following treatment with the methanolic extract of *H. abyssinica* was attributed to its phenolic and flavonoid content [68]. From the molecular docking study, Model Oris could be further investigated through molecular dynamics simulation and clinical trials as an inhibitor of AChE in the treatment of AChE-related disease conditions.

Furthermore, metals and metalloids can induce oxidative stress in neurons, acting as pro-oxidants that increase amounts of lipid peroxide in the brain [69]. The present study revealed significantly elevated levels of MDA and NO in animals exposed to the quaternary mixture, which were effectively reversed by co-administration of the aqueous extract of PA. This counteractive effect on oxidative stress can be ascribed to the extract's rich contents of antioxidant phenolics and flavonoids [70]. Polyphenols have demonstrated the ability to protect rats from metal-induced brain neuroinflammation and cognitive impairments [71],[72].

Metals induce expression of tumor necrosis factor alpha (TNFα), a marker of glial inflammation, in the cerebellum following sub-chronic exposure to mercury [8]. Pro-inflammatory cytokines like TNF-α, IL-1, and apoptotic factor caspase-3 are crucial in neurodegeneration and apoptosis [73],[74]. In vitro and in vivo studies have shown that inorganic arsenic (As) causes oxidative stress, leading to decreased neurite outgrowth and, at higher doses, neuronal cell death [75]. However, co-exposure to antioxidants can alleviate As-mediated oxidative stress [76]–[78]. In this study, treatment with the aqueous extract of PA demonstrated a decrease in pro-inflammatory cytokines, decreased cortical apoptotic factor caspase-3, and the restoration of normal histological appearance in the cerebral cortex and cerebellum compared to rats treated with the quaternary metal mixture alone. These observations are like the restoration of normal expression of the neuronal pro-survival protein Bcl2 and downregulation of the pro-apoptotic protein Bax elicited by *H. abyssinica* of animals treated with AlCl3 may be mediated by the neuroprotective flavonoid and phenolics of Prosopis Africana PA [50],[79].

Nutraceuticals are known to scavenge ROS and ultimately alleviate cellular injury caused by oxidative stress [80],[81]. Additionally, many nutraceuticals possess a pharmacological modulatory potential that can co-activate the Nrf2-ARE antioxidant defense and neurotrophin signaling-mediated cell survival systems [82]–[85]. Therefore, bioactive compounds in nutraceuticals may hold therapeutic potential in the management of neurodegenerative diseases.

In the current study, exposure to the quaternary metal mixture resulted in a significant increase in Nrf2 levels and a significant decrease in hmox-1 levels in the cerebral cortex and cerebellum. However, co-administration of HMM with 1000 mg/kg and 1500 mg/kg of PA extract led to a significant decrease in Nrf2 levels in these brain regions. The activation of the Nrf2-ARE pathway upregulates a network of cooperating enzymes that constitute an antioxidant defense system [86],[87], which maintains redox homeostasis by involving antioxidant synthesis and metabolism and iron homeostasis, including Hmox-1. Hmox-1 overexpression has been observed in neurodegenerative diseases, potentially serving as a compensatory mechanism against oxidative damage [88],[89]. This study has demonstrated remarkable changes in SOD, CAT, and GSH levels following exposure to the metal mixture, which were subsequently reversed by co-treatment with aqueous extract of PA. Natural plants such as *Prosopis africana* are known to display hormetic biphasic dose–response effects, with low-dose aqueous extracts of PA activating or stimulating intracellular Nrf2 antioxidant pathway for neuroprotection to environmental toxicants (heavy metals); at the same time, high doses of polyphenols can be toxic and inhibit these protective pathways. This is in line with the results of the study, indicating that a dose of 500 mg/kg of PA is protective against heavy metals by a significant increase in the Nrf2 level in the cortex and cerebellum of rats, while doses of 1000 or 1500 mg/kg induce a downregulation of Nrf2 levels.

The histopathological findings supported these biochemical parameters, further confirming the potential benefits of PA extract in mitigating neurotoxicity induced by the metal mixture in rats. Collectively, PA extract may exert neuroprotective activity against memory impairment induced by the quaternary metal mixture through the modulation of the oxido-inflammatory response through the Nrf2 signaling pathway. To demonstrate the increased trafficking of Nrf2 from the cytosol to the nucleus, immunoblotting will be done in future studies. Furthermore, the relevant mechanisms behind the decrease in heavy metal content in target organs following treatment with PA should be explored in future studies. Perhaps a weakness of the present study is the lack of mercury data, which will also be addressed in future studies.
